# Dietary rambutan peel powder as a rumen modifier in beef cattle

**DOI:** 10.5713/ajas.19.0342

**Published:** 2019-08-03

**Authors:** Thiwakorn Ampapon, Metha Wanapat

**Affiliations:** 1 Tropical Feed Resources Research and Development Center (TROFREC), Department of Animal Science, Faculty of Agriculture, Khon Kaen University, Khon Kaen 40002, Thailand

**Keywords:** Methane, Plant Secondary Compounds, Rambutan Peel Powder, Rumen Enhancer

## Abstract

**Objective:**

The experiment was conducted to study the effect of rambutan (*Nephelium lappaceum*) fruit peel powder (RP) on feed consumption, digestibility of nutrients, ruminal fermentation dynamics and microbial population in Thai breed cattle.

**Methods:**

Four, 2-year old (250±15 kg) beef bull crossbreds (75% Brahman×25% local breed) were allotted to experimental treatments using a 4×4 Latin square design. Four dietary supplementation treatments were imposed; non-supplementation (control, T1); supplementation of RP fed at 2% of dry matter intake (DMI) (low, T2); supplementation of RP fed at 4% of DMI (medium, T3) and supplementation of RP fed at 6% of DMI (high, T4). All cattle were given a concentrate supplement at 1% of body weight while Napier grass was provided as a free choice.

**Results:**

The findings revealed that RP supplementation did not negatively affect (p>0.05) DMI of Napier grass, while RP intake and total DMI were the greatest in the RP supplementation at 4% and 6% DMI. Nevertheless, the nutrients (dry matter, organic matter, crude protein, neutral detergent fiber, and acid detergent fiber) digestibilities were not changed in the RP supplementation groups. Rumen fermentation parameters especially those of total volatile fatty acids, acetate and butyrate were not significantly changed. However, the propionate concentration was remarkably increased (p<0.05) in the RP supplementation. Notably, the ratio of acetate to propionate, the number of protozoa, as well as the methane estimation were significantly reduced in the RP supplemented groups (4% and 6% of DMI), while the counts of bacteria was not altered.

**Conclusion:**

Supplementation of RP (4% of DMI) improved rumen propionate production, reduced protozoal population and methane estimation (p<0.05) without a negative effect on feed consumption and nutrients total tract digestibilities in beef cattle. Using dietary rambutan fruit peel powder has potential promise as a rumen regulator.

## INTRODUCTION

Russell and Rychlick [1] stated the importance of rumen ecology on rumen fermentation efficiency and the subsequent production of livestock. The use of feed additives such as antibiotics can improve rumen fermentation efficiency. While, research and development regarding methane (CH_4_) production in ruminants have been receiving considerable attention in which mitigation of the rumen CH_4_ has been the main issue [2]. The ruminal methane production is associated with global warming and the loss of digestible energy intake (8% to 12% of total energy intake) [3]. Currently, plants rich in plant metabolites (condensed tannins [CT], saponins [SP]) and essential oils have been receiving more interest regarding their selective inhibition of some rumen microbes and their fermentation [4]. Rambutan (*Nephelium lappaceum*) is a tropical fruit, grown in Southeast Asia e.g. Thailand, Malaysia, Indonesia, Vietnam and eaten fresh and can produce the products such as jams, juice and canned products. Rambutan peel s contains a high level of phenolic compounds such as tannins, SP, flavanoi, garaiin etc [5]. Locally available feed resources containing plant metabolites (polyphenols) especially those of anthrocyanidins and/or SP have been shown to increase the rumen propionate (C_3_), decreased rumen degradation of protein, reducing CH_4_ production and increasing conjugated linoleic acid in ruminant products [6]. Therefore, rambutan peel powder (RP) is a potential supplement to manipulate the rumen process. Nevertheless, modification of rumen fermentation in ruminants by using RP containing CT and SP has been relatively limited. Plant secondary metabolites namely SP and tannins which can impact on rumen microorganisms and fermentation, increased total volatile fatty acid (VFA), supporting the ruminant hosts, whilst mitigating rumen methane production as stated by Poungchompu et al [7]. Hence, this experiment aimed at investigating the influence of RP levels on feed consumption, total tract nutrients digestibilities, rumen fermentation dynamics and methane production in Thai native cattle.

## MATERIALS AND METHODS

### Feed preparation, experimental design of animals

In brief, the RP was prepared as follows; the rambutan peels were collected from fresh fruit peel at Malee Group Public Company Limited in Nakhon Pathom province, Thailand. The peels were sun-dried for about 5 days to attain about 90% DM and ground into powder form. Feed ingredients and their nutritive values are presented in Table 1.

The study was conducted at the Tropical Feed Resources Research and Development Center (TROFREC), Department of Animal Science, Faculty of Agriculture, Khon Kaen University (KKU), Thailand. All procedures involving animals in the metabolism studies were approved by the Institutional Animal Care and Use Committee of Khon Kaen University (KKU) (Ref. no. AEKKU 18/2558).

Four, 2-year old (250±15 kg) crossbred beef cattle (75% Brahman×25% Thai native), were assigned to receive experimental diets in a 4×4 Latin square design. The dietary treatments were as follows; non-supplementation (T1); supplementation of RP fed at 2% of dry matter intake (DMI) (T2); supplementation of RP fed at 4% of DMI (T3) and supplementation of RP fed at 6% of DMI (T4), respectively. The experimental cattle were offered concentrate mixture at 1.0% of body weight (BW), fed two times a day, in the morning and in the afternoon, while Napier grass (Pak Chong I) was fed freely. Mineral blocks and water were provided as a free choice during which the animals were kept in individual pens. This experiment was conducted comprising of the preliminary and the actual feeding regimes for four periods, and each period lasted for 21 days. After the first 14 days, all beef cattle were well adjusted to dietary treatments and samples of the supplement and Napier grass including the left-over were collected, every day during the entire feeding periods. During the last 7 days, feeds and fecal were collected from each animal in the morning before feeding time (07:00 am) samples of feces w in each period. All samples were then composited for each animal period. They were stored in freezer until preparation for chemical analysis.

### Data collection, sample collection, and chemical analyses

Feeds and fecal samples were analyzed for their chemical composition by the method of AOAC [8], neutral detergent fiber (NDF), acid detergent fiber (ADF) according to Van Soest et al [9] and acid-insoluble ash (AIA). The AIA was used as an internal indicator to predict the digestibility of nutrients as described by Van Keulen and Young [10]. Plant secondary metabolites especially proantrocyanidins or CTs were chemically analyzed by the Vanillin-HCL method [11] and SP using methanol extraction as described by Wanapat and Ngamsaeng [12].

Respective rumen fluid from each bull was sampled via stomach tube using vacuum pump to withdraw rumen fluid at 0, 4 h post feeding. The rumen pH and temperature were immediately measured using the measurement meter (HANNA instrument HI 8424 microcomputer, Singapore). Respective samples of rumen fluid from treatments were then thoroughly filtered. The second portion of rumen fluid was then fixed with 10% formalin solution (1:9 v/v, rumen fluid:10% formalin) for total direct counts of microorganisms (bacteria, protozoa, and fungal zoospores) by haemacytometer [13]. Samples were analyzed for rumen VFAs produced from rumen fermentation, for NH_3_-N, where 5 mL of H_2_SO_4_ solution (1 M) was added to 45 mL of rumen fluid. The mixture was centrifuged at 1,600×g for 15 min, and the supernatant was later stored at −20°C prior to VFA analyses using high-performance liquid chromatography [14]. The blood sample from jugular vein, about 10 mL, was collected into tubes with ethylenediaminetetraacetic acid, separated by centrifugation at 500×g for 10 min at 4°C then the plasma was stored at −20°C until later analysis of blood urea N according to the method of Crocker [15]. Details of the experimental protocols used under this experiment were fully reported in Wanapat et al [16].

The VFAs (C _2_, C_3_, C_4_) were used in the equation as described by Moss et al [17] in order to predict the methane estimation.

$${\text{CH}}_{4}\;\text{estimation}=0.45(\text{acetate},{\text{C}}_{2})-0.275(\text{propionate},{\text{C}}_{3})+0.4(\text{butyrate},{\text{C}}_{4})$$

### Statistical analyses

All the data were statistically analyzed using procedure general linear model (Statistical Analysis System [SAS], 2013) [18] according a 4×4 Latin square design. Treatment trends were statistically compared using orthogonal polynomials. The results were presented as mean values with the standard error of the means. Difference among means with p<0.05 was accepted as statistical differences.

## RESULTS

### Experimental ingredients used in diets and their chemical analyzes

The composition of the concentrate, rambutan peel powder and Napier grass, are shown in Table 1. The nutritive values of fibrous components (NDF, ADF), and crude protein (CP) were 184 and 107 and 141 g/kg DM in the concentrate, respectively. Napier grass contained 102, 697, and 435 g/kg DM of CP, NDF, and ADF, respectively. The rambutan peel powder contained 120 and 105 g/kg DM of CTs and SP, respectively.

### Feed consumption and nutrients digestibilities

The DMI measured by kg/d, % BW/d, and g/kg BW^0.75^/d data are presented in Table 2. DM intake of Napier grass and concentrate were similar by RP supplementation. As shown, the DM of Napier grass ranged from 5.7 to 5.8 kg DM/d, while the concentrate intakes were provided as 1% of BW. However, total DMI and DM intake of RP were linearly increased, while CT and SP intake were increased when RP was added at 2%, 4%, and 6% DMI. Furthermore, nutrient digestibilities were not affected by the RP supplementation except at the high level of RP (4% and 6% of DMI).

### Ruminal fermentation, blood, methane estimation, and microbial population

Ruminal fermentation characteristics by supplementation of RP are shown in Table 3. The results of rumen pH (6.6 to 6.7), temperature (39.0°C to 39.8°C) remained unchanged (p> 0.05). Rumen NH_3_-N (17.5 to 18.1 mg/dL) and blood urea nitrogen (BUN) (9.7 to 10.4 mg/dL) were not altered (p>0.05). Furthermore, ruminal fermentation parameters (VFAs, C_2_, C_4_) were not significantly impacted by dietary treatments imposed. However, C_3_ was significantly increased in the RP supplementation at both 4% and 6% of DMI (T3, T4). Moreover, the C_2_:C_3_ ratio and methane estimation were significantly deceased in the supplementation at 4%, 6% of DMI (T3, T4). Nevertheless, the counts of total bacteria were not affected by RP the supplementation whilst, the number of protozoa was significantly reduced with an increased level of RP supplementation, meanwhile the number of fungal zoospores were similar in the RP supplementation groups.

## DISCUSSION

### Feed consumption and digestibilities of nutrients

Supplementation of RP did not change DMI of Napier grass, whilst the total DMI, RP intake, CT intake, and SP intake were linearly increased when RP was added at 2%, 4%, and 6% DMI. However, the lack of effect on apparent digestibility under this experiment could be explained by the low concentration of CT in the supplementation levels used (0%, 2%, 4%, 6% DMI), which RP (6% DMI) contained 0.7% CT and 0.6% SP of DMI, while as reported by Beauchemin et al [19] higher concentrations of CT (>50 g/kg DMI reduces DMI and digestibility. of Poungchompu et al [7], who used a higher level of CT (>3% CT of total DMI), also reported a decrease of DMI and digestibility. This could be attributed to its coating of feed particles and an effect on the cellular membrane of the rumen bacteria.

### Ruminal fermentation, blood, methane estimation, and microbial population

Under this study, the rumen pH, temperature and ruminal NH_3_-N were not affected in the RP supplemented groups. The rumen pH of 6.5 to 6.8 was suitable for the bacterial activity especially cellulolytic bacteria, whilst BUN concentrations did not differ among treatments [20]. Wanapat and Pimpa [21] who stated that rumen degradable protein in the form of rumen NH_3_-N was essentially to be used for rumen microbial protein production efficiency. The optimal concentration of rumen NH_3_-N (15 to 30 mg/dL) required for digestion by microorganisms has been reported when the ruminants were fed on rice straw [20,21].

In this experiment, the ruminal fermentation parameters (total VFA, C_2_, and C_4_) remained similar by the RP supplementation, while the C_3_ production was increased in the RP supplemented groups. It could be due to the relationships with feed intake improvement and the rumen microorganism activity. The ratio of C_2_:C_3_ was reduced as a result of the RP supplementation groups, which agreed with the data of Foiklang et al [22] who revealed that using grape pomace powder (GPP) supplement remarkably increased the total VFA and C_3_ production, while rumen methane estimation was dramatically decreased in the supplemented treatments. Similarly, Gunun et al [23] also reported that C_3_ was increased, while C_2_ and C_4_ were similar when supplementing with plants containing of CT and SP less than 3% of total DMI. Norrapoke et al [24] who found that dietary of mangosteen peel powder (MSP) enhanced total VFA concentration, increasing C_3_, reducing C_2_:C_3_ and methane production in the MSP supplementation in swamp buffaloes.

Under this experiment, the total bacterial and fungal zoo spores were similar among treatments, while the number of protozoa was reduced in the RP supplementation (4% to 6% DMI) groups. Norrapoke et al [24] who used MSP pellet at 300 g/hd/d in dairy cows, found no influence on rumen bacterial and fungal zoospores. Rumen protozoa and methanogens have a symbiotic relationship, in using CO_2_ to produce methane, and the methanogens adhering to the protozoa were reported to be responsible for 9% to 37% of the methane emissions in cattle. Plant metabolic compounds, essentially SP and CTs, have been reported to suppress rumen CH_4_ production [2]. The influence of tannins on methane emission depends on fermentation of microbes and the enzymes secreted [25]. Plant secondary metabolites (CT and SP) have been shown to exert impact on activity of rumen methanogens and protozoa activities that would then limit the H_2_ availability for methanogenesis in the rumen [26]. Furthermore, plant secondary compounds were found to react on the sterols membrane of the protozoa [27]. The possible actions of CT in reducing methane production include the indirect effect on reducing H_2_ formation and the activity of protozoa and methanogens [28]. In the present experiment, the t methane production and protozoal population were subsequently decreased while fungal zoospores were not affected by the RP supplementation. This agrees with Shokryzadan et al [29] who found that supplementation of MSP reduced the rumen microbial population, especially protozoa and methanogens, as well mitigating methane production in ruminants. Paengkoum et al [30] also found that using CTs extracted from mangosteen peel could reduce gas production and concentration of ruminal methane in an *in vitro* gas experiment. Methanogens and CH_4_ production were significantly decreased when the MSP supplementation level was increased, the least value was obtained at 300 g/d. During the rumen fermentation process, enteric CH_4_ is produced when the metabolic hydrogen produced by rumen cellulolytic bacteria reacted with CO_2_ [31]. Bhatta et al [3] explained that tannins considerably suppressed the rumen methane production by decreasing the protozoal population found in an *in vitro* study. While, Foiklang et al [22] discovered that supplementation of GPP could suppress protozoal populations and mitigate methane production in cattle. Similarly, Poungchompu et al [7] revealed that using 4% of MSP as a supplement could decrease rumen protozoa and, hence reduced methane production, accordingly. Currently, Aditya [32] and Gunun et al [23] also reported that using rambutan peel powder at 16 to 20 mg, remarkably decreased methane concentration in *in vitro* gas kinetic experiment. These results reiterate the impact of CTs and SP on rumen fermentation and the end-products.

## CONCLUSION

Under this experiment, it could be summarized that RP supplementation at 4% of the total DMI did not exert any effects on feed intake. However, ruminal propionate concentration was significantly enhanced, while C_2_:C_3_ ratio, methane estimation and protozoal population were dramatically reduced. This study suggested that RP would be a promising dietary rumen enhancer without an adverse effect on feed consumption and nutrients total tract digestibilities.

## Figures and Tables

**Table 1:** Feed ingredients and chemical composition of the experimental diets

Items	Concentrate	RP	Napier grass
Ingredients (g/kg DM)			
Cassava chip	600.0	-	-
Rice bran	100.0	-	-
Coconut meal	80.0	-	-
Palm kernel meal	80.0	-	-
Soybean meal	80.0	-	-
Molasses	15.0	-	-
Urea	30.0	-	-
Mineral mixture	5.0	-	-
Salt	5.0	-	-
Sulfur	5.0	-	-
Chemical composition (g/kg DM)			
Dry matter	877.0	887.0	302.0
Organic matter	926.0	966.0	914.0
Ash	74.0	34.0	86.0
Crude protein	141.0	44.0	102.0
Neutral detergent fiber	184.0	313.0	697.0
Acid detergent fiber	107.0	268.0	435.0
Condensed tannins	-	120.0	-
Saponins	-	105.0	-

RP, rambutan peel powder; DM, dry matter.

**Table 2:** Effect of rambutan peel powder on feed intake and apparent digestibility in beef cattle

Items	Supplementation level of RP (g/kg of DMI)				SEM	Contrasts	
	
	0	2	4	6		L	Q
Napier grass (DM)							
kg/d	5.7	5.8	5.8	5.8	0.03	0.484	0.293
% BW/d	2.1	2.2	2.2	2.2	0.11	0.675	0.256
g/kg BW^0.75^	86.7	87.0	88.2	87.3	0.43	0.612	0.567
Concentrate intake							
kg/d	2.6	2.6	2.7	2.6	0.11	0.328	0.193
% BW/d	1.0	1.0	1.0	1.0	0.05	0.142	0.153
g/kg BW^0.75^	40.1	40.0	40.4	40.2	0.27	0.167	0.351
RP intake							
kg/d	0^a^	0.17^b^	0.34^c^	0.51^d^	0.02	0.012	0.973
% BW/d	0^a^	0.06^b^	0.13^c^	0.19^d^	0.01	0.025	0.703
g/kg BW^0.75^	0^a^	2.6^b^	5.2^c^	7.6^d^	0.05	0.011	0.545
Total DM intake							
kg/d	8.3^a^	8.5^ab^	8.8^b^	8.9^b^	0.03	0.001	0.447
% BW/d	3.1^a^	3.2^a^	3.3^ab^	3.4^b^	0.04	0.002	0.732
g/kg BW^0.75^	126.8^a^	129.6^b^	133.8^c^	135.1^c^	0.41	0.001	0.487
CT intake							
g/d	0.0^a^	20.2^b^	40.1^c^	61.2^d^	2.42	0.012	0.043
% total intake	0.0^a^	0.24^b^	0.47^c^	0.69^d^	0.06	0.001	0.011
SP intake							
g/d	0.0^a^	17.7^b^	35.7^c^	53.6^d^	2.11	0.001	0.021
% total intake	0.0^a^	0.21^b^	0.41^c^	0.61^d^	0.05	0.001	0.001
Apparent digestibility (%)							
Dry matter	65.1	65.7	66.3	64.9	0.52	0.162	0.457
Organic matter	67.1	67.5	67.7	66.9	0.91	0.218	0.312
Crude protein	65.9	66.1	66.7	66.1	0.32	0.112	0.178
Neutral detergent fiber	73.2	73.5	73.1	72.8	0.64	0.231	0.732
Acid detergent fiber	62.7	62.6	63.1	63.0	0.55	0.125	0.435

RP, rambutan peel powder; DMI, dry matter intake; SEM, standard error of the mean; L, linear; Q, quadratic; DM, dry matter; BW, body weight; CT, condensed tannins; SP, saponins.

a–d Means in the same row with different superscripts differ (p<0.05).

**Table 3:** Effect of rambutan peel powder on rumen fermentation and microbial population in beef cattle

Items	Supplementation level of RP (g/kg of DMI)				SEM	Contrasts	
	
	0	2	4	6		L	Q
Ruminal pH	6.7	6.7	6.6	6.6	0.09	0.144	0.221
Ruminal temperature (°C)	39.7	39.8	39.4	39.0	0.14	0.175	0.337
Ruminal NH_3_-N (mg/dL)	17.5	17.7	18.1	17.9	0.06	0.235	0.283
BUN (mg/dL)	9.7	10.3	10.4	10.1	0.11	0.120	0.311
Total VFA (mmol/L)	97.3	99.8	99.3	97.5	1.25	0.243	0.675
VFA (mol/100 mol)							
Acetic acid (C_2_)	69.5	69.0	68.5	69.1	0.20	0.123	0.139
Propionic acid (C_3_)	18.7^a^	18.9^a^	20.9^b^	20.5^b^	0.15	0.013	0.421
Butyric acid (C_4_)	11.8	12.1	10.6	10.4	0.57	0.144	0.111
C_2_:C_3_	3.7^a^	3.7^a^	3.3^b^	3.4^b^	0.06	0.034	0.476
Methane estimation (mM/L)1)	30.9^a^	30.7^a^	29.3^b^	29.6^b^	0.16	0.021	0.121
Total direct counts							
Bacteria (×10^10^ cell/mL)	9.8	10.0	10.5	10.2	0.52	0.198	0.257
Protozoa (×10^5^ cell/mL)	10.5^a^	7.6^b^	5.0^c^	4.2^d^	0.72	0.017	0.143
Fungal zoospore (×10^6^ cell/mL)	2.7	3.1	3.3	3.1	0.44	0.156	0.423

RP, rambutan peel powder; DMI, dry matter intake; SEM, standard error of the mean; L, linear; Q, quadratic; BUN, blood urea nitrogen; VFA, volatile fatty acids.

1) Calculated according to Moss et al [17]. CH_4_ estimation = 0.45(acetate)–0.275(propionate)+0.4(butyrate).

a–d Means in the same row with different superscripts differ (p<0.05).

## References

[1] Russell JB, Rychlik JL (2001). Factors that alter rumen microbial ecology. Science.

[2] Hook SE, Wright ADG, McBride BW (2010). Methanogens: Methane producers of the rumen and mitigation strategies. Arch.

[3] Bhatta R, Sejian V, Gaughan J, Baumgard L, Prasad C (2015). Reducing enteric methane emission using plant secondary metabolites. Climate change impact on livestock: adaptation and mitigation.

[4] Wallace RJ, McEvan NR, McIntosh FM, Teferedegne B, Newbold CJ (2002). Natural products as manipulators of rumen fermentation. Asian-Australas J Anim Sci.

[5] Sun L, Zhang H, Zhuang Y (2012). Preparation of free, soluble conjugate, and insoluble-bound phenolic compounds from peels of rambutan (*Nephelium lappaceum*) and evaluation of antioxidant activities *in vitro*. J Food Sci.

[6] Cieslak A, Szumacher-Strabel M, Stochmal A, Oleszek W (2013). Plant components with specific activities against rumen methanogens. Animal.

[7] Poungchompu O, Wanapat M, Wachirapakorn C, Wanapat S, Cherdthong A (2009). Manipulation of ruminal fermentation and methane production by dietary saponins and tannins from mangosteen peel and soapberry fruit. Arch Anim Nutr.

[8] AOAC (2012). Official Methods of Analysis.

[9] Van Soest PJ, Robertson JB, Lewis BA (1991). Methods for dietary fiber, neutral detergent fiber, and nonstarch polysaccharides in relation to animal nutrition. J Dairy Sci.

[10] Van Keulen J, Young BA (1977). Evaluation of Acid-Insoluble Ash as a Natural Marker in Ruminant Digestibility Studies. J Anim Sci.

[11] Wanapat M, Poungchompu O (2001). Method for estimation of tannin by vanillin-HCL method (A Modified Method of Burns, 1971).

[12] Wanapat M, Ngamsaeng A (2004). Method for estimation of crude saponins (A Modified Method of Kwon et al., 2003).

[13] Galyean M (1989). Laboratory procedure in animal nutrition research.

[14] Samuel M, Sagathewan S, Thomas J, Mathen G (1997). An HPLC method for estimation of volatile fatty acids of ruminal fluid. Indian J Anim Sci.

[15] Crocker CL (1967). Rapid determination of urea nitrogen in serum or plasma without deproteinization. Am J Med Technol.

[16] Wanapat M, Gunun P, Anantasook N, Kang S (2014). Changes of rumen pH, fermentation and microbial population as influenced by different ratios of roughage (rice straw) to concentrate in dairy steers. J Agric Sci.

[17] Moss AR, Jouany JP, Newbold J (2000). Methane production by ruminants: its contribution to global warming. Ann Zootech.

[18] SAS (Statistical Analysis System) (2013). User’s guide: Statistic, Version 93th Edition.

[19] Beauchemin KA, Kreuzer M, O’Mara F, McAllister TA (2008). Nutritional management for enteric methane abatement: a review. Aust J Exp Agric.

[20] Van Soest PJ, Robertson JBA (1985). Laboratory manual for animal science.

[21] Wanapat M, Pimpa O (1999). Effect of ruminal NH3-N levels on ruminal fermentation, purine derivatives, digestibility and rice straw intake in swamp buffaloes. Asian-Australas J Anim Sci.

[22] Foiklang S, Wanapat M, Norrapoke T (2016). *In vitro* rumen fermentation and digestibility of buffaloes as influenced by grape pomace powder and urea treated rice straw supplementation. Anim Sci J.

[23] Gunun P, Gunun N, Cherdthong A (2018). *In vitro* rumen fermentation and methane production as affected by rambutan peel powder. J Appl Anim Res.

[24] Norrapoke T, Wanapat M, Wanapat S (2012). Effects of protein level and mangosteen peel pellets (Mago-pel) in concentrate diets on rumen fermentation and milk production in lactating dairy crossbreds. Asian-Australas J Anim Sci.

[25] Baruah L, Malik PK, Kolte AP, Dhali A, Bhatta R (2018). Methane mitigation potential of phyto-sources from Northeast India and their effect on rumen fermentation characteristics and protozoa *in vitro*. Vet World.

[26] Haque MN (2018). Dietary manipulation: a sustainable way to mitigate methane emissions from ruminants. J Anim Sci Technol.

[27] Goel G, Makkar HPS (2012). Methane mitigation from ruminants using tannins and saponins. Trop Anim Health Prod.

[28] Tedeschi LO, Ramírez-Restrepo CA, Muir JP (2014). Developing a conceptual model of possible benefits of condensed tannins for ruminant production. Animal.

[29] Shokryzadan P, Rajion MA, Goh YM (2016). Mangosteen peel can reduce methane production and rumen biohydrogenation *in vitro*. S Afr J Anim Sci.

[30] Paengkoum P, Phonmun T, Liang JB, Huang XD, Tan HY, Jahromi MF (2015). Molecular weight, protein binding affinity and methane mitigation of condensed tannins from mangosteen-peel (*Garcinia mangostana* L). Asian-Australas J Animal Sci.

[31] Polyorach S, Wanapat M, Cherdthong A, Kang S (2016). Rumen microorganisms, methane production, and microbial protein synthesis affected by mangosteen peel powder supplement in lactating dairy cows. Trop Anim Health Prod.

[32] Aditya S, Köfer J, Schobesberger H The effect of rambutan peel (*Nephelium lappaceum*) as reducing agent on *in vitro* methane production within creating environment friendly farming. Animal hygiene and sustainable livestock production.

